# Lymph Node Dissection in Testicular Cancer: The State of the Art and Future Perspectives

**DOI:** 10.1007/s11912-024-01511-y

**Published:** 2024-03-02

**Authors:** Bartosz Małkiewicz, Gabriela Świrkosz, Wojciech Lewandowski, Katarzyna Demska, Zuzanna Szczepaniak, Jakub Karwacki, Wojciech Krajewski, Tomasz Szydełko

**Affiliations:** 1 Department of Minimally Invasive and Robotic Urology, University Center of Excellence in Urology, Wrocław Medical University, Wrocław, Poland; 2 University Center of Excellence in Urology, Wrocław Medical University, Wrocław, Poland

**Keywords:** Testicular cancer, Retroperitoneal lymph node dissection, Lymphadenectomy, Retroperitoneal lymph nodes, Lymph node metastasis

## Abstract

**Purpose of Review:**

This narrative review provides a comprehensive overview of the evolving role of retroperitoneal lymph node dissection (RPLND) in the management of testicular cancer (TC). It explores the significance of RPLND as both a diagnostic and therapeutic tool, highlighting its contribution to accurate staging, its impact on oncological outcomes, and its influence on subsequent treatment decisions.

**Recent Findings:**

RPLND serves as an essential diagnostic procedure, aiding in the precise assessment of lymph node involvement and guiding personalized treatment strategies. It has demonstrated therapeutic value, particularly in patients with specific risk factors and disease stages, contributing to improved oncological outcomes and survival rates. Recent studies have emphasized the importance of meticulous patient selection and nerve-sparing techniques to mitigate complications while optimizing outcomes. Additionally, modern imaging and surgical approaches have expanded the potential applications of RPLND.

**Summary:**

In the context of TC management, RPLND remains a valuable and evolving tool. Its dual role in staging and therapy underscores its relevance in contemporary urological practice. This review highlights the critical role of RPLND in enhancing patient care and shaping treatment strategies, emphasizing the need for further research to refine patient selection and surgical techniques.

## Introduction

Testicular cancer (TC) stands as the predominant solid neoplasm among males aged 15 to 40, exhibiting diverse incidence rates among different racial cohorts [1, 2]. TC is relatively rare, accounting for 1% of male tumors and 5% of urological malignancies [3–6]. Over the recent decades, the incidence of TC has risen for unknown reasons, with significant variations among countries [1, 5, 7]. TC falls into two primary categories: germ cell and stromal carcinomas. Germ cell tumors are the most prevalent, constituting 95% of cases, and are further divided into two histopathological subtypes: approximately 55–60% are seminomatous germ cell tumors (SGCTs), and 40–45% are nonseminomatous germ cell tumors (NSGCTs) [8].

TC primarily metastasizes via the lymphatic system, with drainage to retroperitoneal lymph nodes (LNs), including lumbar, celiac, superior, and inferior mesenteric LNs, in 88% of cases [8–13]. However, the primary metastatic site is the inguinal region [14, 15]. Retroperitoneal lymph node dissection (RPLND) is a crucial component of the treatment algorithm for select TC patients. Its role and indications have evolved for both low-stage and advanced TC due to high cure rates achieved by surgery [16]. RPLND conventionally serves as the primary intervention for low-stage NSGCT, encompassing stages IA, IB, and IIA NSGCT, as well as for addressing residual retroperitoneal masses following chemotherapy or as a salvage surgery [17]. Although the surgical approach remains consistent in these instances, the underlying rationale and subsequent outcomes may exhibit variability [18].

The objective of this review is to furnish a comprehensive survey of lymphadenectomy in TC management, encompassing its evolving role in low-stage and advanced cases. Additionally, we delve into contemporary and prospective approaches and therapies, shedding light on the future of TC treatment.

## Data Acquisition

For the sake of this narrative review, we performed a thorough literature search in the English language, reviewing original articles, meta-analyses, systematic reviews, and narrative reviews available in the PubMed database up until May 2023. We conducted searches utilizing diverse combinations of the following terms: testicular cancer; lymphadenectomy; retroperitoneal lymph node dissection; sentinel nodes; lymph node metastasis; nodal staging; robot assisted; open surgical; laparoscopic; complications; risk factors; imaging; surgical approaches. A total of 820 pertinent articles were identified, and after the selection process, the final number of papers included in this manuscript amounted to 315. Studies possessing the utmost level of evidence and relevance to the addressed subjects (185) were chosen, with the concurrence of the authors.

## Anatomical and Surgical Aspect of Lymphadenectomy

### Anatomy of Lymph Drainage

The lymphatic drainage associated with the testicular region is primarily based on retroperitoneal LNs. At the onset of the twentieth century, researchers demonstrated the primary role of LNs adjacent to the great vessels (vena cava inferior, abdominal aorta, common iliac arteries) in the lymphatic drainage of the testes [19]. Further advancements in diagnostic methods have enabled the identification of specific groups of LNs to which lymph from the testes drains [20]. The main drainage in the lymphatic system from the left testicle occurs toward the preaortic, paraaortic, left external iliac, and left common iliac LNs, with subsequent drainage to the precaval, paracaval, interaortocaval, right external iliac, and right common iliac LNs [20]. For the right testis, the primary lymphatic drainage occurs to the precaval, paracaval, interaortocaval, preaortic, right external iliac, and right common iliac LNs, with subsequent drainage to the para-aortic, left external iliac, and left common iliac LNs [20]. The difference in drainage in the lymphatic system between the left and right testicles affects the frequency of metastasis occurrence in LNs. Metastases predominantly occur in ipsilateral LNs to the affected organ [21, 22]. In the left TC, the frequently affected LNs are the preaortic, paraaortic, interaortocaval, left common iliac, and testicular vessel zones. In the right TC, the commonly affected LNs are the aortocaval, precaval, paracaval, preaortic, paraaortic, right common iliac, and right testicular vessels [20–29]. Metastases rarely involve regions above the renal hilum. In some instances, contralateral LNMs can occur, even in 20% of stage II TC patients [20–22, 28, 29].

### Surgical Techniques and Lymphadenectomy Templates

RPLND is a fundamental treatment approach for TC, serving as primary therapy (P-RPLND) for early-stage tumors or salvage post-chemotherapy (PC-RPLND). Recent advancements [30–33] have refined surgical techniques and lymphatic drainage understanding, leading to the definition of LN dissection templates. Initially, RPLND encompassed the renal hilum and LNs along the major vessels, but due to high complications and infrequent LN involvement, the procedure has evolved to exclude the renal hilum region [20–22, 28, 29, 34, 35].

**Fig. 1:**
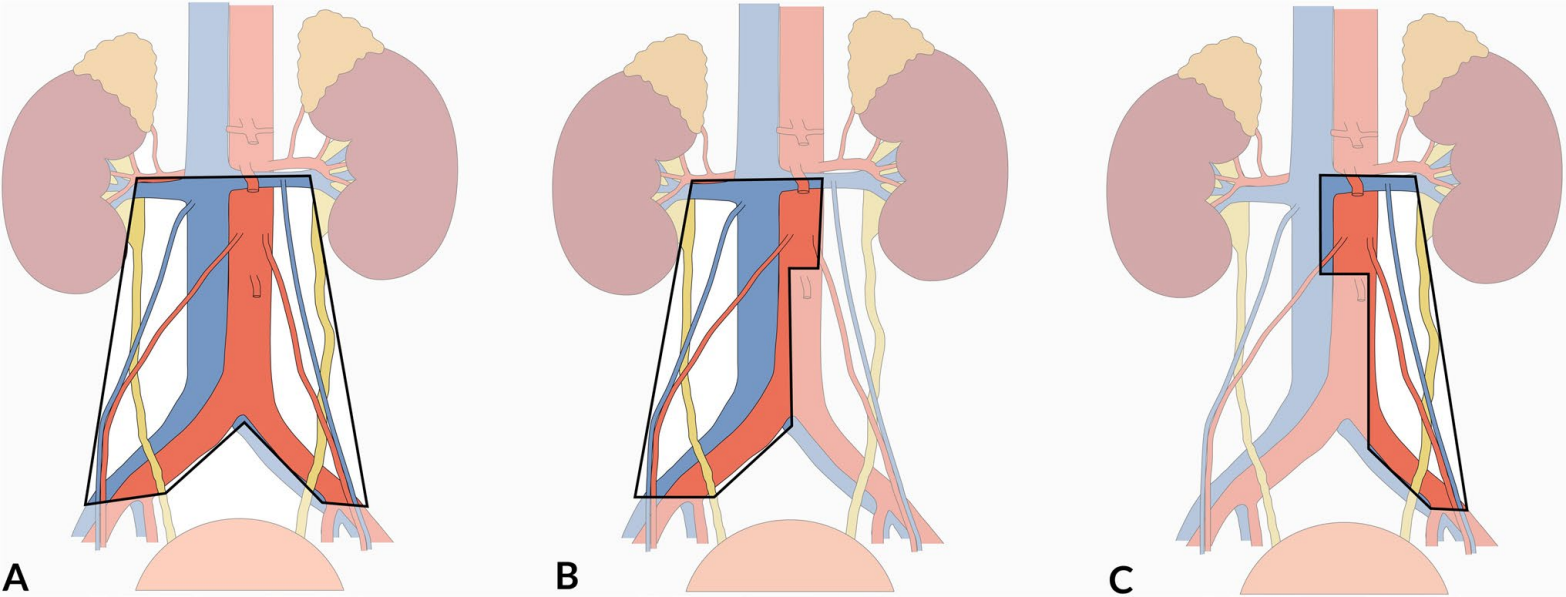
Anatomical extent of lymphadenectomy in testicular cancer. **A** Bilateral template. **B** Right unilateral template. **C** Left unilateral template

The essential RPLND template covers the area below the renal vessels and extends to encompass both sides of the common iliac and the proximal one-third of the external iliac regions. It includes paracaval, precaval, interaortocaval, preaortic, paraaortic, ipsilateral and contralateral iliac, and gonadal vein LNs. This template forms the foundation of RPLND techniques. Controversies arise due to its extent and complications related to retrograde ejaculation, leading to various modifications. These modifications have resulted in unilateral RPLND templates, including the right template (precaval, paracaval, interaortocaval, preaortic, right common iliac, and right gonadal regions) and left template (paraaortic, preaortic, interaortocaval, left common iliac, and left gonadal regions) [36–40]. The anatomical templates of lymphadenectomy are depicted in Fig. 1.

Jewett and Donohue introduced the nerve-sparing technique, offering the potential for better long-term oncological outcomes and reduced complications by preserving radicality during RPLND [32, 33]. In this approach, surgeons identify and dissect postganglionic sympathetic nerve fibers responsible for ejaculation. These fibers run along the dorsal surface of the aorta to the superior hypogastric plexus, just below the inferior mesenteric artery (IMA). Right fibers are found in the aortocaval zone, while left fibers often course near the paraaortic nodal packet [32, 33, 41–44]. This technique can be applied in both unilateral and bilateral templates and is highly effective, preserving antegrade ejaculation in over 90% of cases [32, 33, 45].

Moreover, there have been notable advancements in the field of minimally invasive RPLND (miRPLND) techniques in recent years. Presently, RPLND procedures encompass open surgery (oRPLND), laparoscopy (lRPLND), and more recently, robotic-assisted techniques (rRPLND) [30, 31]. In oRPLND, the patient is positioned supine, and a midline abdominal incision is executed. To access the retroperitoneum, the posterior peritoneum is incised along the small bowel mesentery root, extending from the caecum to the ligament of Treitz. The split and roll technique is then implemented, commencing below the left renal vein crossings along the aorta and progressing downward to locate the IMA origin. In bilateral templates, the IMA is ligated and divided, while in unilateral templates, it is preserved. To reduce complications, lymphatic channels are ligated. Improved LN access is achieved by ligating lumbar arteries. Subsequently, gonadal and lumbar veins are ligated, facilitating LN harvesting [41]. rRPLND utilizes the da Vinci robotic system and involves two approaches. In the transperitoneal flank approach, the patient is placed in a lateral position, and the docking robotic system is performed over the patient’s shoulder or flank area. Placements of ports are strategically determined, either in the midline or on the side of the dissection. To gain access to the retroperitoneum, an incision is performed along the Toldt’s white line, and the colon is medially reflected. This approach ensures effective access to the affected side and spermatic cord. Another approach is the supine transperitoneal approach, where the patient lies supine and is positioned in steep Trendelenburg. Docking the robotic system is held over the patient’s head, with ports positioned diagonally in the lower abdomen, oriented towards the side of the affected testicle. Exposure is achieved by making an incision on the posterior peritoneum up to the ligament of Treitz. Notably, this technique enables performing bilateral templates without the need for redocking [30, 46]. Further insights into minimally invasive and contemporary surgical methodologies are expounded upon in the “Modern Surgical Approaches” section.

### Complications

Surgical procedures, including RPLND, carry inherent risks of complications. Some of these complications are related to direct intervention in the retroperitoneal region. During RPLND, there is a risk of damaging nerve fibers responsible for ejaculation and encountering complications associated with lymphatic trauma [47–51]. Moreover, there is a possibility of injuring major blood vessels or nearby organs [47, 48, 51]. The mortality rate associated with RPLND is relatively low, ranging from 0.27 to 0.48% in studies involving large cohorts [52–54]. However, patients with advanced disease and multiple risk factors may experience severe complications, leading to systemic instability and, in some instances, death [51–54].

The incidence of complications fluctuates based on the type of RPLND performed. PC-RPLND has a higher complication rate (ranging from 14 to 30%), compared to P-RPLND (7 to 24%) [52, 55–60]. Minimally invasive techniques, including laparoscopic and robotic approaches, show fewer complications than open methods [61–66]. Notably, there are no statistically significant differences in complication rates between lRPLND and rRPLND [67–69]. Several studies have reported reduced complication rates with the unilateral template compared to the bilateral template [38, 59, 70–72]. Complications are more common in patients over 40 years old and in cases involving tumors larger than 20 mm [72]. The correlation between the quantity of harvested LNs and the incidence as well as the severity of complications is evident, specifically in those categorized as Clavien-Dindo grade 3 or higher [16].

**Fig. 2:**
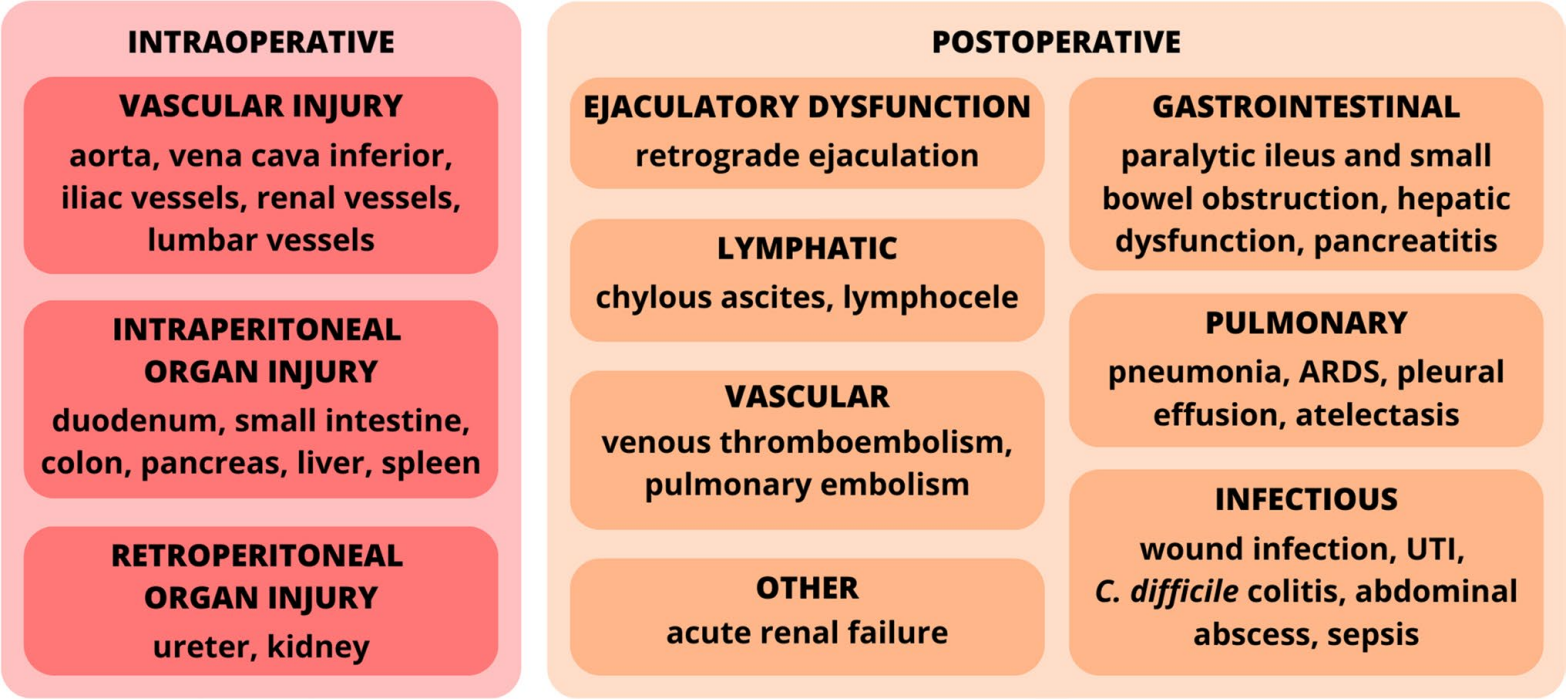
An overview of intra- and postoperative complications of lymphadenectomy in testicular cancer management. ARDS, acute respiratory distress syndrome; UTI, urinary tract infection; *C. difficile*, *Clostridium difficile*

Retrograde ejaculation and its results are significant concerns for RPLND patients. The implementation of the nerve-sparing technique in RPLND substantially reduces this complication [55]. Many centers report excellent outcomes, with over 93% preservation of antegrade ejaculation [16, 38, 45, 55, 71, 73–75]. Some studies suggest that a unilateral template may yield better results in preserving antegrade ejaculation than a bilateral one [38, 69, 76]. However, in PC-RPLND with advanced tumors, nerve-sparing techniques are often impractical, resulting in poorer outcomes, with less than 70% preservation of antegrade ejaculation in such cases [52]. Figure 2 depicts the most common complications of the lymphadenectomy.

## Prognostic Factors for Nodal Involvement

The identification of prognostic biomarkers in testicular cancer assumes paramount significance in the context of clinical management, as it not only facilitates a deeper understanding of disease progression but also holds the potential to inform more precise risk stratification, therapeutic decision-making, and prognostication of patient outcomes [77].

In a study involving 353 TC patients who underwent orchiectomy from 1993 to 2009, 90 cases with over 30% embryonal carcinoma underwent P-RPLND. Their analysis revealed a significant link between endovascular invasion, embryonal carcinoma, and retroperitoneal metastatic risk. Patients in stage II had significantly more LNMs than stage I, indicating that higher-stage TC poses a greater risk of retroperitoneal LN metastasis [78•].

**Fig. 3:**
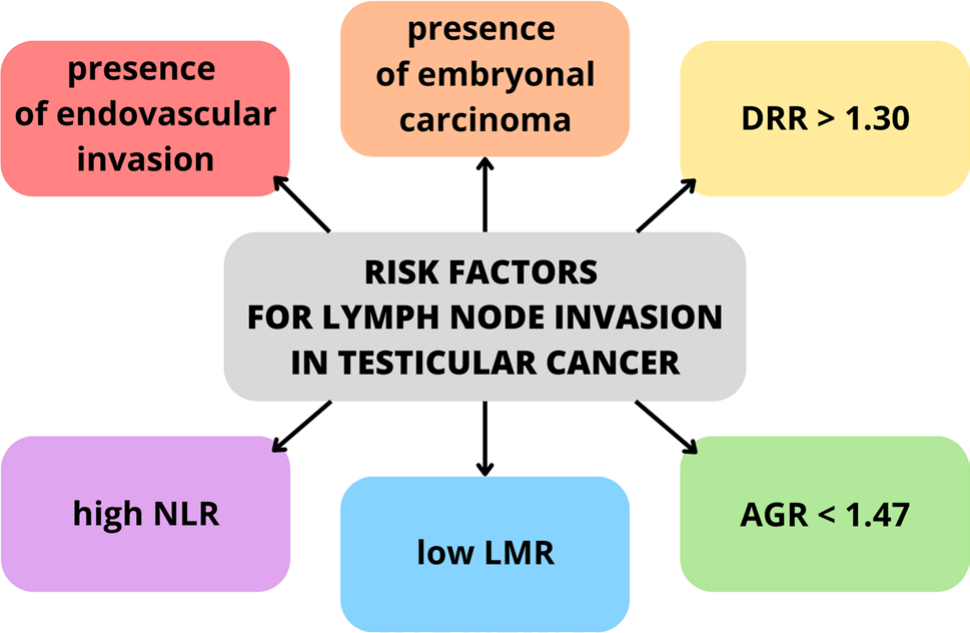
Modern risk factors for metastasis in lymph nodes in testicular cancer. AGR, albumin-globulin ratio; DRR, de Ritis ratio; NLR, neutrophil-to-lymphocyte ratio; LMR, lymphocyte-to-monocyte ratio

In a retrospective study from March 2007 to January 2017, 45 TC patients with LNMs and 73 without were analyzed. They explored the aspartate aminotransferase to alanine aminotransferase ratio (De Ritis ratio (DRR)) as a predictor. Results showed that a DRR score exceeding 1.30 preoperatively may independently predict retroperitoneal LNM and organ metastasis, influencing treatment decisions [79•]. In another study involving 99 radical orchiectomy patients, the DRR value was inconclusive. Researchers examined five factors: neutrophil-to-lymphocyte ratio (NLR), lymphocyte-to-monocyte ratio (LMR), platelet-to-lymphocyte ratio (PLR), neutrophil-to-monocyte ratio (NMR), and DRR. Only NLR and LMR proved significant, with higher NLR and lower LMR correlating with advanced-stage cancer, metastasis, and retroperitoneal LN invasion [80]. In a different retrospective analysis of 115 patients who underwent radical inguinal orchiectomy between 2007 and 2018, researchers focused on the preoperative albumin to globulin ratio (AGR). They found that AGR < 1.47, along with lymphovascular invasion, predicted retroperitoneal nodal and distant metastasis. AGR was significantly lower in deceased patients, making it a useful survival prognosticator [77]. Figure 3 presents an overview of contemporary risk factors associated with metastasis in LNs in TC.

LND is one of the treatment methods in TC, but it is also an important diagnostic tool and should be always performed with the aim of removing potential metastases in lymph nodes [81, 82]. Higher number (> 40) of LNs obtained during surgery gives a bigger possibility of finding metastases in the probe and improving diagnostic efficiency of the procedure [83]. LND gives a chance to confirm the presence or absence of neoplasm cells in lymph nodes and change the preoperative TNM stage based on that. It is a base to adapt methods of treatment to individual patient [82].

## Therapeutic Role of LND in TC

### Oncological Outcomes

An analysis of 20-year-long data from patients who underwent P-RPLND or PC-RPLND showed overall survival (OS) rate of 89%, cancer-specific survival (CSS) rate of 92%, and recurrence-free survival (RFS) of 85%. During a 120-month follow-up, 15% of these patients experienced recurrence [84]. In another study, during a 33-month follow-up, 9% of patients experienced relapse after lRPLND or rRPLND [85••]. For patients who underwent primary rRPLND, 4% experienced relapse in an 8-month follow-up, which is consistent with previous studies reporting 2-year RFS rates of 91% and 97%, respectively [86–88].

In patients with NSGCT, PC-RPLND may be performed. Previous studies reported recurrence rates in open bilateral PC-RPLND ranging from 0 to 22.7% [76, 89–100] and in open unilateral PC-RPLND ranging from 3 to 40% [90, 92, 93, 95, 98, 99, 101–105]. Bilateral PC-RPLND showed an 80% OS at 38-month follow-up [91] and a 5-year disease-specific survival (DSS) rate of 74% [106] and 98% [107], respectively. Unilateral modified template PC-RPLND showed a 75.5% OS at 47-month follow-up [108], 99% OS at 10-year follow-up, 93% RFS after 5 years, and 92% RFS after 10 years [102].

Regarding bilateral post-chemotherapy minimally invasive retroperitoneal lymph node dissection (PC-miRPLND), several studies reported recurrence rates ranging from 0 to 10% [71, 89, 93, 109, 110], while unilateral PC-miRPLND resulted in recurrence rates ranging from 0 to 10.5% [71, 74, 89, 93, 104, 109–115]. Some studies on bilateral and unilateral post-chemotherapy robotic-assisted retroperitoneal lymph node dissection (PC-rRPLND) reported 0% recurrence rates [116–120]. However, a recent study [72] showed a 20.6% recurrence rate in patients who underwent PC-rRPLND, while other studies [121–123] reported relapse rates of 4.65%, 6.7%, and 8%, respectively. The limited research and varying results in this area indicate a need for further studies to explore possible mediators of these outcomes.

### Impact on Further Therapeutic Process

RPLND can be used as a diagnostic tool to determine appropriate treatment for individuals with TC. A group of NSGCT patients who undergo low-volume nodal metastasis resection at RPLND were evaluated in terms of predictive factors for relapse. Individuals with persistent marker elevation were significantly more likely to suffer a relapse than those with normal markers, sufficiently managed by observation alone. Primary chemotherapy should be advised especially to patients who had elevated markers before RPLND [124]. These findings correspond with further data [125].

In a 2004 study, 99% of patients with stage II NSGCT, who received adjuvant etoposide and cisplatin chemotherapy after P-RPLND, did not experience a relapse in an 8-year follow-up. With proven effectiveness, the authors are certain that it should be offered to pN2 NSGCT patients [126], whereas pN1 NSGCT patients were found to benefit from RPLND only [127]. Furthermore, bilateral nerve-sparing lRPLND performed on stage I, stage IIA marker-negative, and post-chemotherapy stage IIB patients proved no retroperitoneal recurrence at a mean follow-up of 17.2 months [128], which stands in line with other studies regarding the efficacy of lRPLND in pN + patients [129]. Additionally, lRPLND followed by adjuvant chemotherapy, with two cycles of bleomycin, etoposide, and cisplatin, resulted in no recurrence in pN + patients on a mean follow-up of 84 months [73]. Another research showed that 56 out of 58 patients with stage I NSGCT, who received chemotherapy consisted of 2 cycles of cisplatin, vinblastine (or etoposide), and bleomycin, remained relapse-free on a median follow-up of 93 months [130].

**Fig. 4:**
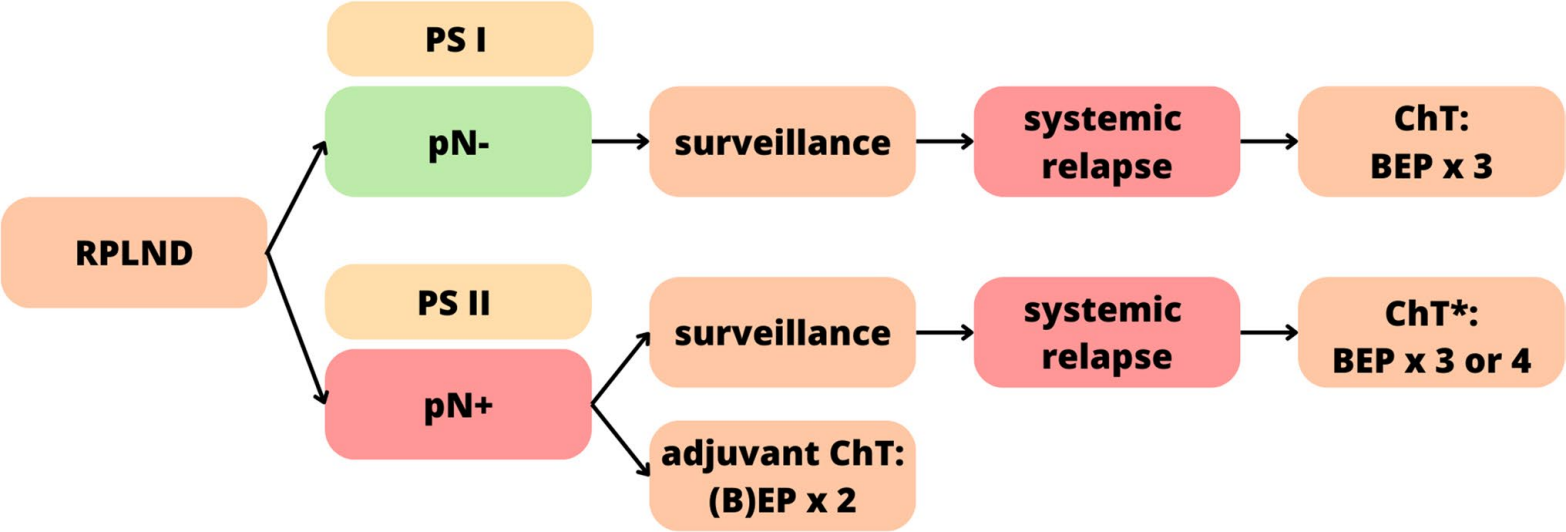
The guidelines provided by the European Association of Urology on the treatment decision-making process after lymphadenectomy in testicular cancer. RPLND, retroperitoneal lymph node dissection; PS, pathologic stage; pN, pathologic lymph node staging; ChT, chemotherapy; BEP, bleomycin, etoposide, cisplatin. *After systemic relapse in pN + patients, standard chemotherapy is indicated

The European Association of Urology (EAU) guidelines suggest either adjuvant chemotherapy or surveillance after RPLND [131]. These guidelines are overviewed in Fig. 4.

## Guidelines

EAU guidelines for RPLND in TC state that RPLND should be performed by an experienced surgeon in a referral center. Nerve-sparing RPLND should only be offered to stage IB (pT2-pT4) NSGCT patients with contraindications to adjuvant chemotherapy and unwilling to undergo surveillance. In addition, it may be considered as an initial treatment in stage IIA NSGCT patients without elevated tumor markers. Men with postpubertal teratomas with a somatic malignant component may also benefit from P-RPLND and should be advised to consider it [131].

The American Urological Association (AUA) indicates RPLND as an alternative treatment option for patients with stage IA NSGCT who decline surveillance or are at risk for noncompliance. According to the AUA, RPLND should be proposed to patients with stage IB NSGCT and is recommended for patients with stage I NSGCT and teratoma with malignant transformation in the primary tumor at the time of orchiectomy. It is also recommended for individuals with stage IIA NSGCT with normal serum (S0) alpha-fetoprotein (AFP) and human chorionic gonadotropin (hCG) levels after orchiectomy [132].

The European Society for Medical Oncology (ESMO) recommendations are consistent with the previous ones and emphasize the importance of strict selection of stage I NSGCT patients for nerve-sparing oRPLND. ESMO suggests nerve-sparing P-RPLND in stage IIA patients with negative markers and a single progressive LN. In addition, patients in post-chemotherapy management with residual LNs larger than 1 cm in axial diameter should undergo nerve-sparing oRPLND [133]. Table 1 provides a summary of the EAU, the AUA, and ESMO recommendations on this topic.

**Table 1:** Overview of indications for RPLND in TC according to the guidelines provided by the EAU, the AUA, and the ESMO

Guidelines	Indications for RPLND	References
EAU	• Nerve-sparing RPLND should be offered to stage IB NSGCT patients unwilling to undergo surveillance or with contraindications to adjuvant chemotherapy, and to stage IIA NSGCT patients without elevated tumor markers	[131]
	• P-RPLND should be recommended for men with postpubertal teratoma with a somatic malignant component	
AUA	• RPLND should be proposed to all patients with stage IB NSGCT, patients with stage IA NSGCT who are unwilling to undergo surveillance, and patients with stage IIA NSGCT with normal AFP and hCG after orchiectomy	[132]
	• Patients with stage I NGSCT and teratoma with malignant transformation in the primary tumor at orchiectomy may be selected for RPLND	
ESMO	• RPLND is generally not recommended in stage I NSGCT	[133]
	• Residual LNs with an axial diameter greater than 1 cm should be removed by nerve-sparing oRPLND in NSGCT patients afterward	

*EAU*, The European Association of Urology; *AUA*, The American Urological Association; *ESMO*, The European Society for Medical Oncology; *RPLND*, retroperitoneal lymph node dissection; *TC*, testicular cancer; *NSGCT*, non-seminomatous germ cell tumor; *AFP*, alpha-fetoprotein; *hCG*, human chorionic gonadotropin; *LNs*, lymph nodes

## Future Perspectives

### Preoperative Nodal Staging

#### Conventional Imaging

The National Comprehensive Cancer Network (NCCN) guidelines for TC advise the conduction of a CT scan of the abdominal and pelvic regions with intravenous contrast for all individuals who receive a diagnosis of either seminoma or NSGCT [134, 135]. CT scans are highly sensitive in detecting LNs thanks to their outstanding spatial resolution. However, they are unable to definitively distinguish between benign and cancer-invaded LNs, especially in case of smaller nodes [8, 136].

To identify suspicious LNs, short-axis size criteria are commonly employed, but the specific cutoff values may vary among different medical centers and specialists. Several studies have investigated the most suitable short-axis LN size cutoff as an indicator of neoplastic involvement, using RPLND as the reference standard [137–140]. When using a threshold of 10 mm or more to identify involved LNs, CT exhibits excellent specificity (> 90%) but limited sensitivity (37–47%) [136, 137]. Reducing the threshold to 4 mm enhances sensitivity to 93% but decreases specificity to 58% [136]. In general, LNs are considered suspicious when their maximum short-axis diameter measures 8–10 mm or more [141]. Employing this CT size cutoff for retroperitoneal LNs yields a highly significant area under the curve (AUC), with both sensitivity and specificity nearing 70% [137].

At present, CT remains the initial imaging modality for surveillance to assess retroperitoneal LNs [9, 142, 143]. Baessler et al. carried out a research investigation [144] to assess the capability of CT radiomics in improving the prognostication of malignant histopathology in retroperitoneal lymph node metastases (LNM) from NSGCTs before post-chemotherapy retroperitoneal lymph node dissection (PC-RPLND). Their discoveries can be condensed as follows: (i) utilizing a gradient-boosted tree model trained on the five most vital CT radiomic features led to a diagnostic sensitivity/specificity of 88%/78%; (ii) a classifier relying solely on “size” criteria produced a moderate diagnostic sensitivity/specificity of 64%/68%; and (iii) the incorporation of the radiomics classifier would have notably decreased surgical overtreatment by 46% in the independent test and validation groups. This proposed strategy should be amalgamated with established clinical biomarkers and subjected to additional validation through extensive prospective clinical trials.

MRI and CT are often comparable in their ability to assess LNs during TC staging, and they share similar limitations [8, 10, 76, 145–147]. Both imaging techniques rely on size criteria and cannot definitively distinguish between benign and cancer-invaded LNs based on tissue characteristics. However, the utilization of MRI in TC staging is limited due to its high cost, lengthy process, and a shortage of physicians experienced in interpreting MRI results [8, 141]. Nonetheless, MRI can prove valuable in specific situations, such as when patients have allergies preventing the use of CT or when CT scans produce inconclusive results. Additionally, it may be preferred by young patients who are concerned about radiation exposure [8, 131, 141]. Some authors have suggested a more focused and concise MRI technique that specifically targets retroperitoneal lymph nodes, excluding inguinal and pelvic areas. This approach shortens the acquisition duration from roughly 30–35 min to just 12–13 min [9, 11]. These recommendations align with guidance provided by ESMO and the Swedish and Norwegian Testicular Cancer Group (SWENOTECA), which endorse the utilization of contrast-enhanced CT scans for the initial staging and recommend employing retroperitoneal MRI for post-initial treatment follow-up to mitigate radiation exposure in individuals with TC [9, 148–150].

18F-Fluorodeoxyglucose (FDG) positron emission tomography/computed tomography (PET/CT) has been investigated in various TC scenarios, encompassing the initial staging, post-treatment assessment, and recurrence [134, 151]. Some authors have explored the potential of 18F-FDG PET/CT in the staging of NSGCT [152–157]. However, it has not exhibited efficacy, and according to NCCN guidelines, its utilization is not advised either in the primary setting or following chemotherapy in cases of NSGCT [14]. Another setting in which 18F-FDG PET/CT has been studied is when relapse occurs following definitive TGCT treatment [14, 158]. Ultimately, it remains uncertain whether 18F-FDG PET/CT offers superior diagnostic capabilities compared to CT scans and tumor markers in cases of suspected recurrence in TGCT [134]. The diagnostic and prognostic effectiveness of 18F-FDG PET/CT was also assessed in 114 patients with suspected recurrence of TGCT [159]. The study revealed that 18F-FDG PET/CT achieved a sensitivity of 86.8% and a specificity of 90.2%. Nonetheless, the study did not directly compare it with other imaging modalities, thus leaving it uncertain whether 18F-FDG PET/CT outperforms conventional imaging [134].

Woldu SL et al. evaluated the potential of anti-1-amino-3-18F-fluorocyclobutane-1-carboxylic acid (18F-fluciclovine) PET/CT for the accurate detection of residual NSGCT prior to RPLND [160]. 18F-Fluciclovine enables cancer detection through the modulation of mechanisms that control elevated amino acid uptake, a characteristic prevalent in malignant tumors. This mechanism differs from that of 18F-FDG. In May 2016, the Food and Drug Administration (FDA) granted approval for the utilization of 18F-fluciclovine in a PET/CT scan for men with suspected recurrence of prostate cancer [160–162]. However, the experimental 18F-fluciclovine PET/CT exhibited a low sensitivity of 29% and specificity of 33% when compared to the reference standard of RPLND [160].

#### Novel Imaging Techniques

Lymphotropic nanoparticle MRI (LNMRI) utilizing ferumoxtran-10 has been investigated as a prospective technique for identifying retroperitoneal LNs in individuals with TGCTs. These nanoparticles aggregate within LNs, making them visible on MRI. In LNMRI, abnormal LNs appear as nodules with a mixed signal, featuring an intensified central area surrounded by a peripheral signal decrease. A pilot study by Harisinghani et al. [163] researched LNMRI for identifying hidden metastatic lesions in a cohort of 18 men with TC. LNMRI demonstrated enhanced sensitivity (88% vs. 71%) and specificity (92% vs. 68%) in contrast to traditional MRI or CT imaging methods. [134]. Notably, LNMRI achieved a sensitivity of 100% in detecting positive LNs smaller than 10 mm, which might be missed by conventional imaging methods. Nevertheless, these promising findings remain devoid of external validation owing to the limited sample size and the lack of randomization in the research [9].

The ghrelin receptor, also identified as the growth hormone secretagogue receptor 1a (GHS-R1a), exhibits varying expression in both healthy tissues and multiple malignancies, encompassing prostate, testicular, and ovarian cancers. Researchers are striving to develop ghrelin analogs with enhanced stability and reduced molecular weight that could contain the PET isotope [164]. In chemical studies, this innovative PET radiotracer has demonstrated a strong binding affinity for the ghrelin receptor, with an overall radiochemical yield of 3.1% [134].

Intraoperative imaging holds great promise for identifying viable LNMs during RPLND, ensuring thorough removal of affected LNs. One FDA-approved contrast agent for this purpose is indocyanine green (ICG), which has been extensively studied in diverse urologic malignancies to assist in achieving comprehensive tumor excision and directing lymph node dissection [165–167]. Near-infrared fluorescence (NIRF) technology enables selective in vivo imaging of different tissues according to their natural absorption and reflection characteristics. Through intravenous administration of fluorescent compounds, we can evaluate tissue perfusion or target cell membrane ligands, which become fluorescent upon activation [134].

While reports of ICG-guided surgery for testicular cancer are limited, a preclinical model of RLND by Penna et al. [168] demonstrated that NIRF imaging enhanced the retrieval of lymph nodes compared to the unassisted method. Furthermore, typical RPLND intermittently excised tissue did not match lymphatic tissue on final pathology. In a recent case study, intravenous ICG was employed to aid in the dissection and eliminating a solitary recurrence of seminoma in the left external iliac LN. Over a 6-year follow-up, no indications of a local recurrence were found or distant metastatic spread, without requiring additional treatment. In addition to portraying the LNs, ICG images also offer real-time visualization of the lymphatic drainage route [14]. The researchers observed that NIRF intraoperative imaging allowed for improved delineation of tumor boundaries and aided in the safe removal of the tumor without damaging adjacent structures nearby [134, 158].

Another intraoperative method involves radiotracers emitting single photons and portable gamma imaging devices. Large-field gamma cameras create 3D hybrid images by integrating volumetric Single-Photon Emission Computed Tomography (SPECT) molecular images with CT anatomical images. This allows for accurate detection and localization of LNs in the retroperitoneum, including sentinel LNs, as radiotracers injected into these nodes are absorbed by lymphatic channels [134]. In a study by Zarifmahmoudi et al. [25], nine candidates for post-chemotherapy retroperitoneal lymph node dissection (PC-RPLND) underwent intraoperative SLN mapping. Patients received an injection of 99mTc-nanocolloid into the spermatic cord stump, and LN radioactivity was measured approximately 1.5 h later. In all patients, a full bilateral RPLND was conducted, encompassing the elimination of any residual masses. In six out of nine patients, an intraoperative gamma probe successfully identified one or more SLNs. In two of the nine patients in whom SLNs were successfully detected, pathological analysis indicated metastatic infiltration in both the sentinel lymph node SLN and additional peritoneal LNs that were excised. The investigations conducted by Blok et al. [25] and Zarifmahmoudi L et al. [25] did not report any false-negative detected SLNs, and there were no nodal recurrences during follow-up. This SLN mapping technique appears to be both feasible and promising.

### Modern Surgical Approaches

#### Robotic-Assisted RPLND

The initial encounter with rRPLND was documented in 2006 by Davol et al. [30], and subsequent investigations have been conducted ever since [119, 144, 169–173]. Robotic surgery presents various potential benefits, such as enhanced three-dimensional visual clarity, tremor reduction, and increased range of motion. Ge et al. [174••] compared rRPLND with non-robotic RPLND (NR-RPLND) and observed the following outcomes favoring rRPLND: reduced hospitalization duration, decreased estimated blood loss, and a lower incidence of complications. Nevertheless, in the comparison of rRPLND with oRPLND/lRPLND, comparable outcomes were noted regarding operative duration, lymph node involvement, and postoperative ejaculatory function impairment [174••]. To mitigate the detrimental consequences of radio/chemotherapy, numerous prospective surgical cohorts have been established to explore the potential of RPLND as a therapeutic choice for stage II seminoma [175]. At present, two comprehensive reports have been issued for RPLND series (PRIMETEST, SEMS), whereas investigations from other studies (COTRIMS, Royal Marsden) have solely been presented in the form of abstracts at medical congresses [101, 176, 177]. Additionally, the upcoming publication will also help define the role of miR371 in selecting men with pN + disease, in addition to assessing the oncological efficacy of P-RPLND [178]. The Royal Marsden trial is the only prospective trial to combine adjuvant carboplatin with P-RPLND. The results of these two trials will have implications for the utilization of rRPLND in patients with seminoma, given that a significant proportion of individuals, up to 15%, manifest with metastatic disease. In general, three approaches to rRPLND are described in the literature: flank transperitoneal, supine transperitoneal, and supine extraperitoneal. Each approach has its inherent constraints, and there is presently inadequate clinical data to substantiate the supremacy of any single approach over the remaining options.

The transperitoneal flank approach is the most longstanding robotic method of entry in the TC context, initially outlined by Davol et al. [30]. Within this procedure, the patient is placed in a lateral flank orientation on a contoured table. Several port placements have been delineated for this method, conventionally involving 3–4 robotic ports and 1–2 auxiliary ports situated on the dissection side or midline. The robotic system is subsequently positioned and secured above the patient’s shoulder or flank. Exposure of the retroperitoneal space is accomplished by creating an incision along the Toldt’s white line and medially to the colon. As needed, a hepatic retractor can be employed on the right flank to enhance the visual field. This technique offers sufficient entry to the involved side of the retroperitoneum and spermatic cord. Nonetheless, in situations necessitating a bilateral template, this method may entail the need for repositioning the robot [118, 170], although instances of single docking have also been documented [17, 179].

The supine transperitoneal approach is gaining popularity and has recently been reported by several research groups [17, 119, 170]. In this method, the patient is arranged in a supine orientation and tilted into a steep Trendelenburg position to promote the cranial descent of the bowel. The robotic system is then placed either in a superior position (Si system) or laterally (Xi system). The robotic ports are inserted at an oblique angle into the lower abdominal region, directed towards the lateral aspect of the affected testis. The procedure commences with a posterior peritoneal cut that extends to the Ligament of Treitz, revealing the retroperitoneal space. To facilitate comprehensive dissection of the LNs according to a standard template, the small intestine is suspended from the abdominal wall with a monofilament suture threaded through a straight needle [17, 118]. However, when using the Xi platform, lateral docking is possible, and a complete bilateral template can be dissected with the excision of the remnant cord without the need for redocking [17, 119]. However, when using the Xi platform, lateral docking is possible, and full bilateral template can be meticulously dissected, incorporating the excision of the remnant cord, all without necessitating robot repositioning [17, 119].

Pooleri et al. devised an innovative method for RPLND through the utilization of the da Vinci Xi system in the supine orientation, referred to as robot-assisted supine extraperitoneal retroperitoneal lymph node dissection (RASE-RPLND) [180]. This approach has been reported in a solitary case involving a 31-year-old individual diagnosed with post-chemotherapy NSGCT. The patient was positioned in a supine posture with the addition of a minor sandbag support beneath the right side of the pelvis. Three robotic ports and one working port were introduced via a 3-cm incision positioned in the anterosuperior region of the anterior superior iliac spine. The Xi system was affixed from the opposite side. Extraperitoneal dissection was initiated at the psoas muscle’s outer surface and proceeded anteriorly toward the mass. Pneumatic pressure was used to displace the peritoneal sac anteriorly, providing excellent space for dissection without extensive retraction. The overall surgical duration amounted to 240 min, with an estimated blood loss (EBL) of 60 ml. The individual recovered postoperatively without complications. The authors highlighted several advantages of the supine approach, including early postoperative recovery, improved physiologic airway pressure during prolonged anesthesia, and the capability to execute the procedure without bowel manipulation. Moreover, this technique reduces position-related complications and facilitates emergency resuscitation.

#### Open Midline Extraperitoneal Retroperitoneal Lymphadenectomy (EP-RPLND)

An innovative approach to RPLND incorporates a midline incision that is entirely situated in the extraperitoneal space [181, 182]. Kim et al. [182] initiated this strategy with the aim of diminishing the perioperative and long-term complications linked to peritoneal access. The procedure is initiated with a midline abdominal incision spanning from a point a few centimeters beneath the xiphoid process (approximately corresponding to the level of the renal hilum) to 4–5 cm below the umbilicus (approximating the level of the ipsilateral common iliac artery). Commencing from the infraumbilical segment of the incision, where the peritoneal separation from the fascia is facilitated, the anterior and posterior rectus fascial layers are excised, and the extraperitoneal region between the peritoneum and the transversalis fascia is meticulously expanded through a combination of gentle blunt and sharp dissection techniques. The peritoneal sac is subsequently gently displaced medially, separating it from the inferolateral abdominal wall on the side of the intended dissection, and repositioned toward the ipsilateral psoas muscle [181]. In a recent study, 69 patients underwent EPRPLND employing this midline surgical approach, with 68 of them effectively undergoing the extraperitoneal technique. The authors noted the restoration of gastrointestinal motility by postoperative day 2, accompanied by a median hospital stay of 3 days. The median calculated blood loss amounted to 325 ml, and there were no instances of ileus documented. A total of 12 (17.6%) complications were observed in 11 patients during the 90-day postoperative period [181]. Furthermore, safeguarding the peritoneal sac additionally mitigates inconspicuous fluid seepage, which holds particular significance in patients undergoing bleomycin therapy post-chemotherapy. By refraining from peritoneal cavity penetration, the potential for adhesive intestinal obstruction and small bowel obstruction (SBO) may be reduced, while also preserving the integrity of extraperitoneal sympathetic neural networks.

## Conclusions

In conclusion, RPLND is a valuable tool in the management of TC, both diagnostically and therapeutically. It serves as an essential staging procedure, allowing accurate assessment of LN involvement and facilitating tailored treatment strategies. Therapeutically, RPLND plays a critical role in improving oncological outcomes and survival rates, particularly in patients with certain risk factors and disease stages. It should be considered especially in patients with stage IB and IIA disease without elevated tumor markers. While its impact may vary depending on the specifics of each case, it remains an important option for patients who can benefit from its curative potential. In addition, RPLND influences the subsequent therapeutic process by guiding decisions on adjuvant treatments, such as chemotherapy, and helping to identify patients who may require closer monitoring. Its ability to provide a comprehensive assessment of nodal status and inform subsequent management underscores its continued relevance in the era of modern medicine.

## Data Availability

No datasets were generated or analysed during the current study.

## Abbreviations

^18^F-fluciclovine: Anti-1-amino-3-18F-fluorocyclobutane-1-carboxylic acid
AFP: Alpha-fetoprotein
AGR: Albumin to globulin ratio
AUA: The American Urological Association
AUC: Area under the curve
ChT: Chemotherapy
CSS: Cancer-specific survival
CT: Computed tomography
DRR: De Ritis ratio
DSS: Disease-specific survival
DWI: Diffusion-weighted imaging
EAU: European Association of Urology
EBL: Estimated blood loss
EP-RPLND: Extraperitoneal retroperitoneal lymph node dissection
ESMO: The European Society for Medical Oncology
FDA: The Food and Drug Administration
FDG: ^18^F-Fluorodeoxyglucose
GHS-R1a: Growth hormone secretagogue receptor 1a
hCG: Human chorionic gonadotropin
ICG: Indocyanine green
IMA: Inferior mesenteric artery
LMR: Lymphocyte to monocyte ratio
LN: Lymph node
LND: Lymphadenectomy
LNM: Lymphatic node metastasis
LNMRI: Lymphotropic nanoparticle magnetic resonance imaging
lRPLND: Laparoscopic retroperitoneal lymph node dissection
miRPLND: Minimally invasive retroperitoneal lymph node dissection
MRI: Magnetic resonance imaging
NCCN: The National Comprehensive Cancer Network
NIRF: Near-infrared fluorescence
NLR: Neutrophil to lymphocyte ratio
NMR: Neutrophil to monocyte ratio
nr-RPLND: Non-robotic retroperitoneal lymph node dissection
NSGCT: Nonseminomatous germ cell tumor
oRPLND: Open retroperitoneal lymph node dissection
OS: Overall survival
PC-miRPLND: Post-chemotherapy minimally invasive retroperitoneal lymph node dissection
PC-RPLND: Post-chemotherapy retroperitoneal lymph node dissection
PC-rRPLND: Post-chemotherapy robotic-assisted retroperitoneal lymph node dissection
PET/CT: Positron emission tomography/computed tomography
PLR: Platelet to lymphocyte ratio
P-RPLND: Primary retroperitoneal lymph node dissection
RASE–RPLND: Robot-assisted supine extraperitoneal retroperitoneal lymph node dissection
RFS: Recurrence-free survival
RPLND: Retroperitoneal lymph node dissection
rRPLND: Robotic-assisted retroperitoneal lymph node dissection
SBO: Small bowel obstruction
SGCT: Seminomatous germ cell tumor
SLN: Sentinel lymph node
SPECT: Single-photon emission computed tomography
SWENOTECA: The Swedish and Norwegian Testicular Cancer Group
TC: Testicular cancer
TGCT: Testicular germ cell tumor
UICC: The International Union Against Cancer
